# Caveolin-1/Endothelial Nitric Oxide Synthase Interaction Is Reduced in Arteries From Pregnant Spontaneously Hypertensive Rats

**DOI:** 10.3389/fphys.2021.760237

**Published:** 2021-11-09

**Authors:** Jéssica A. Troiano, Simone R. Potje, Murilo E. Graton, Emily T. Gonçalves, Rita C. Tostes, Cristina Antoniali

**Affiliations:** ^1^Programa de Pós-graduação Multicêntrico em Ciências Fisiológicas, SBFis, São Paulo State University (UNESP), Araçatuba, Brazil; ^2^Department of Basic Sciences, School of Dentistry, São Paulo State University (UNESP), Araçatuba, Brazil; ^3^Department of Physics and Chemistry, Ribeirão Preto, Faculty of Pharmaceutical Sciences of Ribeirão Preto, University of São Paulo (USP), Ribeirão Preto, Brazil; ^4^Department of Biological Sciences, Minas Gerais State University (UEMG), Passos, Brazil; ^5^Department of Pharmacology, Ribeirão Preto Medical School, University of São Paulo (USP), Ribeirão Preto, Brazil

**Keywords:** pregnancy, spontaneously hypertensive rat, aorta - thoracic, mesenteric artery, endothelial nitric oxide synthase, caveolae, caveolin-1

## Abstract

We have investigated the role caveolae/caveolin-1 (Cav-1) plays in endothelial nitric oxide synthase (eNOS) activation and how it impacts pregnancy-induced decreased vascular reactivity in normotensive (Wistar rats) and spontaneously hypertensive rats (SHR). Wistar rats and SHR were divided into non-pregnant (NP) and pregnant (P). Nitrite levels were assessed by the Griess method in the aorta and mesenteric vascular bed. In functional studies, arteries were incubated with methyl-β-cyclodextrin (dextrin, 10mmol/L), which disrupts caveolae by depleting cholesterol, and concentration-response curves to phenylephrine (PE) and acetylcholine (ACh) were constructed. Electronic microscopy was used to determine endothelial caveolae density in the aorta and resistance mesenteric artery in the presence of vehicle or dextrin (10mmol/L). Western blot was performed to evaluate Cav-1, p-Cav-1, calmodulin (CaM), and heat shock protein 90 (Hsp90) expression. Cav-1/eNOS interaction in the aorta and mesenteric vascular bed was assessed by co-immunoprecipitation. Nitric oxide (NO) generation was greater in arteries from P groups compared to NP groups. Dextrin did not change vascular responses in the aorta from P groups or the number of caveolae in P groups compared to NP groups. Compared to NP Wistar rats, NP SHR showed smaller number of caveolae and reduced Cav-1 expression. Pregnancy did not alter Cav-1, CaM, or Hsp90 expression in the aorta or mesenteric vascular bed from Wistar rats or SHR. These results suggest that pregnancy does not alter expression of the main eNOS regulatory proteins, but it decreases Cav-1/eNOS interaction. Reduced Cav-1/eNOS interaction in the aorta and mesenteric vascular bed seems to be an important mechanism to increase eNOS activity and nitric oxide production in pregnant normotensive and hypertensive rats.

## Introduction

Caveolae are invaginations of the plasma membrane of cells, including endothelial cells, and they consist mainly of cholesterol, sphingolipids, and proteins (Severs, 1981; Yao et al., 2009), such as caveolin (Cav) isoforms. Three types of caveolin have been identified in mammalian cells: Cav-1, Cav-2, and Cav-3 (Okamoto et al., 1998; Shaul and Anderson, 1998). Cav-1 is an essential protein in structural caveolae formation, and it is involved in different signal transduction pathways (Razani and Lisanti, 2001; Patel et al., 2008). In endothelial cells, Cav-1 interacts directly with endothelial nitric oxide synthase (eNOS), keeping eNOS inactive by preventing it from interacting with calmodulin (CaM). Under conditions where specific stimuli increase intracellular calcium, CaM recognizes calcium ions and is activated, binding to and activating eNOS (García-Cardeña et al., 1996; Ju et al., 1997). Due to the Cav-1 inhibitory function on eNOS activity, Cav-1 knockout mice show greater eNOS activity in endothelial cells (Razani et al., 2001). On the other hand, CaM (Forstermann et al., 1991) and heat shock protein 90 (Hsp90) (García-Cardeña et al., 1998) positively regulate eNOS activity.

Hsp90 is a chaperone protein present in eukaryotic cells. Hsp90 association with other proteins regulates cell signaling pathways (Pearl and Prodromou, 2006). Hsp90 recruitment by vascular endothelial growth factor, histamine, and shear stress activates eNOS, increasing nitric oxide (NO) production (García-Cardeña et al., 1998; Russell et al., 2000). eNOS activation by Hsp90 causes endothelium-dependent relaxation of blood vessels (García-Cardeña et al., 1998), whereas inhibition of Hsp90 binding to eNOS reduces NO production (Shah et al., 1999). In addition, Hsp90 appears to increase CaM affinity for eNOS and to promote dissociation between eNOS and Cav-1 (Takahashi and Mendelsohn, 2003; Sessa, 2004).

Pregnancy in normotensive Wistar rats and spontaneously hypertensive rats (SHR) is a physiological process characterized by significant reduction in mean arterial pressure despite increased plasma volume. This phenomenon stems from reduced peripheral vascular resistance associated with decreased pressor responses and reduced contractile response to vasoconstrictors (Barron et al., 1984; Chu and Beilin, 1993a,b; Ballejo et al., 2002; Elias et al., 2008; Stennett et al., 2009; Ognibene et al., 2012;Zancheta et al., 2015; Troiano et al., 2016). Compared to blood vessels from non-pregnant rats, eNOS activity is increased in systemic arteries from late pregnant normotensive and hypertensive rats. Increased eNOS activity, and hence greater NO production, negatively modulates contraction of systemic blood vessels during pregnancy (Zancheta et al., 2015; Troiano et al., 2016). In this study, we hypothesized that pregnancy changes cell signaling involving caveolae, Cav-1, CaM, Hsp90, and eNOS in arteries from normotensive and SHR. No study has analyzed potential alterations in these proteins, caveolae density, or Cav-1/eNOS interaction in arteries from pregnant hypertensive rats. Given that Cav-1, CaM, and Hsp90 control eNOS activity, this study aimed to analyze the role caveolae and Cav-1 play in eNOS modulation and vascular reactivity of arteries from pregnant normotensive and hypertensive rats.

## Materials and Methods

The Animal Use Ethics Committee of the School of Dentistry of Araçatuba (CEUA-FOA/UNESP) approved all the experiments in this study (protocol n° 2015-00730).

### Animals

Female normotensive Wistar rats and SHR (systolic blood pressure – SBP≥150mmHg) aged 12weeks were divided into non-pregnant (NP Wistar rats and NP SHR) and pregnant (P Wistar rats and P SHR). NP groups were studied in the estrous phase of the estrous cycle, and P groups were studied at the end of pregnancy (18–20th day of pregnancy). For mating, three female rats and one male rat of the same strain (Wistar rats or SHR) were housed in the same box during the night. Day zero of pregnancy was determined by the presence of sperm in the morning vaginal smear. Animals received standard food and water *ad libitum*, and they were kept under controlled temperature (22–24°C) and humidity (45–65%) with light–dark cycles [12-h (hours) light/12-h dark]. SBP was measured by the Tail-Cuff Plethysmography method (PowerLab, ADInstruments, Melbourne, Australia).

### Nitrite Level Determination

The Griess reaction, which converts nitrate to nitrite, was used to determine nitrite levels as a NO metabolite (Green et al., 1982) in thoracic aortic and mesenteric vascular bed homogenates. The Griess reagent (50μl), consisting of sulfanilamide (1%, w/v), naphthylethylenediamine dihydrochloride (0.1%, w/v), and orthophosphoric acid (25%, v/v), was added to the homogenates (50μl) and incubated at room temperature for 10min (minutes). Absorbance was measured in a spectrophotometer at 540nm and compared to known concentrations of a sodium nitrite curve (0–200μmol/l). Nitrite levels in the samples were normalized to the protein content of the respective thoracic aortic or mesenteric vascular bed samples. To know the magnitude of the increase in nitrite levels in the blood vessels from P Wistar rats and P SHR, the variation (Δ) of nitrite levels was calculated; that is, nitrite levels in P Wistar rats or P SHR were subtracted from nitrite levels in NP Wistar rats or NP SHR, respectively. The results are expressed in μmol/L/μg of protein.

### Vascular Reactivity

Thoracic aortic rings (2mm) were placed between two stainless steel hooks and connected to an isometric force transducer (DMT, ADInstruments, Melbourne, Australia), maintained in a chamber containing Krebs solution (mmol/L): NaCl 130.0, KCl 4.7, KH_2_PO_4_ 1.2, MgSO_4_ 1.2, NaHCO_3_ 14.9, glucose 5.5, and CaCl_2_ 1.6; pH 7.4; 95% O_2_ and 5% CO_2_; 37°C. Rings were stabilized for 30min at a baseline tension (30 mN), and their vitality was confirmed with high concentration of potassium chloride (KCl, 120mmol/L). Endothelium integrity was confirmed by acetylcholine-induced relaxation (ACh, 10μmol/L) in rings pre-contracted with phenylephrine (PE, 1μmol/L). Rings that showed relaxation responses to ACh above 70% in SHR and above 90% in Wistar rats were considered to have endothelium. Concentration-response curves to PE and ACh (0.1nmol/L to 100μmol/L) were plotted in the presence of vehicle or methyl-β-cyclodextrin (dextrin, 10mmol/L). Data are expressed as maximum effect (E_max_) of PE contraction or ACh relaxation, pD_2_ (negative logarithm transformation of EC_50_, the concentration that produced half-maximal contraction or relaxation amplitude), and area under the curve (AUC), in arbitrary units (AUC represents the magnitude of vasoconstriction or vasodilation).

### Electron Microscopy

The aorta and resistance mesenteric artery were dissected and incubated with vehicle (control) or dextrin (10mmol/l) for 60min, as previously described (Moreira et al., 1996). Arteries rings were fixed by immersion in a solution containing glutaraldehyde (2%) and paraformaldehyde (2%) in sodium cacodylate buffer (0.1mmol/L) for 24h. Preparations were kept in sodium cacodylate (0.1mmol/L), in a freezer, until the next step. Sample infiltration was performed with Araldite resin and pure acetone, combined at different ratios, for 24h. Next, the material was polymerized with Araldite resin at 60°C for 72h. Plastic blocks were trimmed, and 0.5μm semi-thin sections (Leica ultra-microtome, Wetzlar, Germany) were stained with toluidine blue (1%) so that appropriate areas could be selected for ultrathin sectioning (60–70nm). Ultrathin sections were collected on carbon-coated single-slot grids followed by contrast with uranyl acetate and lead citrate. Electron micrographs were taken at an initial magnification of 10,000×. Micrographs were photographically enlarged to 50,000× on the computer screen so that details of the caveolae could be visualized. Morphometry and quantitative analysis were performed with the ImageJ software (National Institutes of Health, Bethesda, MD, United States). Caveolae were counted in the peripheral cytosolic space next to endothelial cell membranes. The mean of the groups represents the caveolae count in five different images of each rat divided by the number of rats in each group (*n*=4–5). Results are expressed as the number of caveolae/μm^2^ in the endothelial cytoplasm, representing the caveolae density.

### Western Blot

The thoracic aorta and mesenteric vascular bed were removed from Wistar rats and SHR, dissected, and stored at −80°C. Tissues were macerated in liquid nitrogen and homogenized in modified RIPA buffer and protease inhibitor with a sonicator (Vibra Cell Sonics, Newtown, United States). Homogenates were centrifuged (10,000rpm, 4°C, 20min), and protein concentration in the supernatant was determined by the Lowry method (Lowry et al., 1951). Bovine serum albumin was used as standard. Sixty micrograms (60μg) of total protein was applied for electrophoresis on 10% polyacrylamide gel and transferred to a nitrocellulose membrane. Membranes were blocked with skimmed milk (5%) at room temperature for 1h. Membranes were incubated with primary antibodies for anti-Cav-1 (610,406, BD Biosciences, United States) 1:2000, anti-p-Cav-1 (611,338, BD Biosciences, United States) 1:1000, anti-CaM (sc-137,079, Santa Cruz Biotechnology, United States) 1:250, anti-Hsp90 (610,418, BD Biosciences, United States), and anti-β-actin (A5441, Sigma-Aldrich, United States) at 4°C overnight. Then, membranes were incubated with anti-mouse secondary antibodies at room temperature for 1h. Bands were detected with chemiluminescent substrate for peroxidase and visualized with ImageQuant LAS 500 (GE Healthcare Life Sciences, Little Chalfont, United Kingdom). β-actin was used to normalize results. Band intensity was quantified with the optical densitometry software ImageJ. Results are in arbitrary units.

### Co-immunoprecipitation

The co-immunoprecipitation assay was performed according to the manufacturer’s protocol (Protein A/G PLUS-Agarose, sc-2003, Santa Cruz, United States) and as already performed by our group (Troiano et al., 2021). Briefly, a pool (*n*=3) of thoracic aorta and resistance mesenteric bed (three samples per pool) was lysed in octyl-D-glucoside (ODG, 2%) buffer. Supernatant proteins (500μg) were rotated with anti-eNOS antibody (610297, BD Biosciences, United States) at 4°C overnight, which was followed by rotation with protein A/G PLUS-agarose beads at 4°C for 3h. Then, samples were washed in Tris-buffer six times. Next, proteins were eluted with Laemmli sample buffer. After that, samples were boiled at 100°C for 10min, so eNOS and Cav-1 were detected by Western blot. Results are expressed, in arbitrary units, by the ratio between the intensities of Cav-1 expression and immunoprecipitated eNOS expression.

### Statistical Analysis

Results are expressed as the mean±standard error of the mean of obtained values, and *n* indicates the number of rings, arteries, mesenteric vascular bed, or pools used in each group and experiment.

After Shapiro–Wilk normality tests, results were compared between groups by using two-way ANOVA, followed by the *post hoc* Tukey test. To calculate the variation (Δ) of nitrite levels, results were compared between groups by using Student’s *t*-test. GraphPad Prism software version 6.0 (GraphPad Software Corporation, La Jolla, CA, United States) was employed. Differences were considered significant when *p*<0.05.

## Results

### Pregnancy Increases Nitrite Levels in the Aorta and Mesenteric Vascular Bed From Wistar Rats and SHR

**Figure 1:**
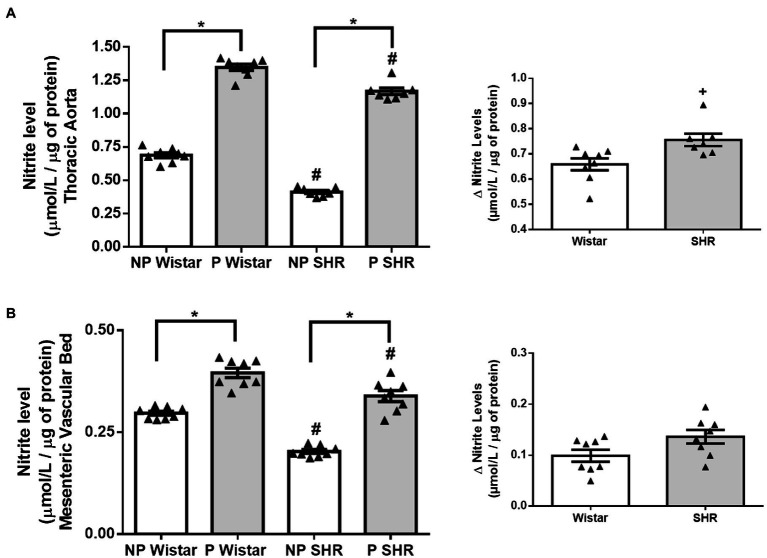
Nitrite levels (μmol/L/ug of protein) and calculation of variation (Δ) of nitrite levels in aortic **(A)** and mesenteric vascular bed **(B)** of non-pregnant (NP) and pregnant (P) Wistar and spontaneously hypertensive (SHR) rats. The data represent the mean±SEM of the results (*n*=7–8). ^*^p<0.05 P versus NP groups; ^#^*p* <0.05 NP SHR and P SHR versus NP Wistar rats and P Wistar rats; + *p* <0.05 SHR versus Wistar rats.

Compared to NP Wistar rats, nitrite levels in aorta and mesenteric vascular bed homogenates from NP SHR were lower (Figures 1A,B). Pregnancy increased nitrite levels in the thoracic aorta, and this increase had greater magnitude in Wistar rats than in SHR. (Figure 1A). In mesenteric vascular bed homogenates, pregnancy increased nitrite levels in Wistar rats and SHR similarly (Figure 1B, *p*= 0.0543).

### Aorta From Pregnant Rats Resists the Effects of Dextrin on PE and ACh Reactivity

**Figure 2:**
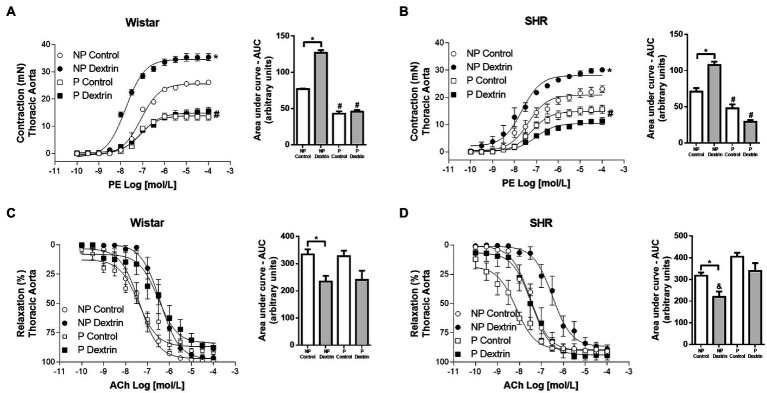
Concentration-effect curves (0.1nmol/L to 100μmol/L) to phenylephrine (PE, in **A,C**) and acetylcholine (ACh, in **B,D** ) in aortic rings from non-pregnant (NP) and pregnant (P) Wistar and spontaneously hypertensive (SHR) rats in the presence of vehicle or dextrin (10mmol/l). From the concentration-effect curves, we calculated the area under the curve (AUC). The data represent the mean±SEM of the results (*n*=5). ^*^p<0.05 E_max_/AUC NP dextrin versus NP control; ^#^*p*<0.05 E_max_/AUC P control and P dextrin versus NP control and NP dextrin; ^&^AUC NP dextrin versus P control and P dextrin.

We evaluated the effect of dextrin, a cholesterol-depleting agent that disrupts caveolae, on vascular reactivity to PE and ACh and compared these effects for the aorta from NP Wistar rats, P Wistar rats, NP SHR, and P SHR. Dextrin increased PE E_max_ and AUC, in the aorta from NP Wistar rats, but PE potency remained unchanged. Interestingly, dextrin did not alter PE E_max_, AUC, or potency in P Wistar rats (Figure 2A). PE-induced contraction and AUC increased in the presence of dextrin in the aorta from NP SHR, but this drug did not modify PE potency (Figure 2B). As in the case of the aorta from P Wistar rats, dextrin did not significantly change PE E_max_, AUC, or pD_2_ in aortic rings from P SHR (Figure 2B).

Dextrin did not change ACh E_max_ or potency in the aorta from NP Wistar rats or P Wistar rats compared to the respective controls (Figure 2C). The same observation was true for the aorta from NP SHR and P SHR (Figure 2D). Although these parameters did not change, dextrin promoted relaxation of smaller magnitude in the aorta from NP Wistar rats and NP SHR compared to the respective NP control curves, while the magnitude of relaxation to ACh remained unaltered in P Wistar rats and P SHR (Figures 2C,D – AUC).

### Hypertension, Pregnancy, and Dextrin Reduced the Number of Caveolae in the Aorta From Rats

**Figure 3:**
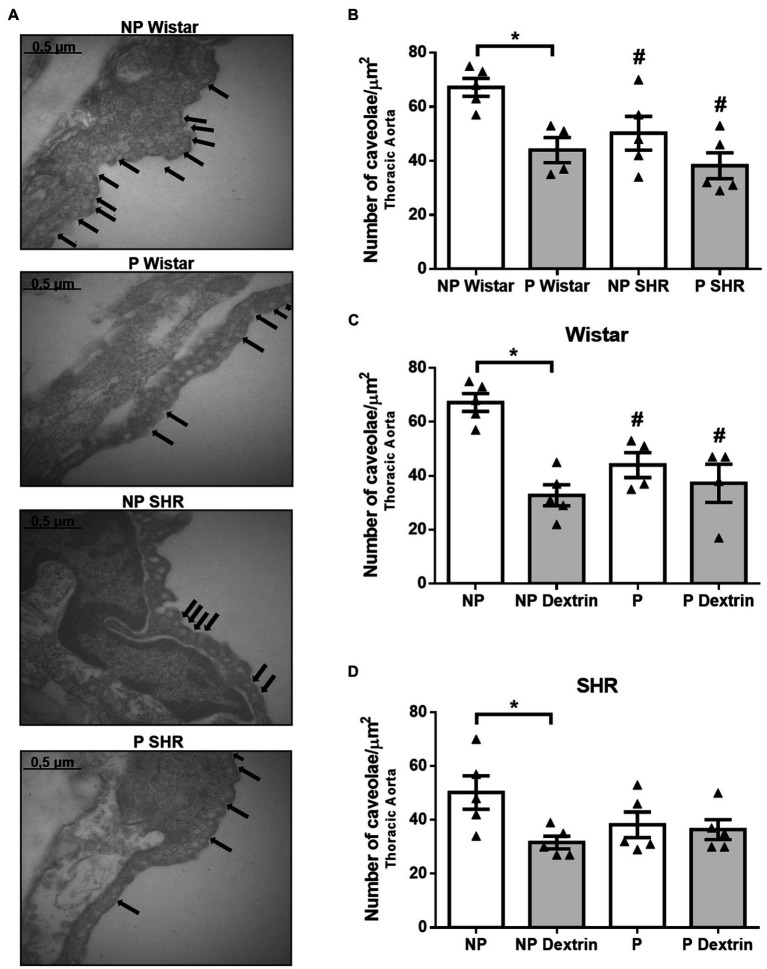
Representative photomicrographs (50,000× magnification, in **(A)** and quantification of the number of endothelial caveolae/μm^2^ in aorta of non-pregnant (NP) and pregnant (P) Wistar and spontaneously hypertensive (SHR) rats **(B)** in the presence of vehicle or dextrin (10mmol/L, **C,D**). The bars represent the mean±SEM of the results obtained in the aorta (*n* =4–5) from the different groups. ^*^p <0.05 P Wistar rats or P dextrin versus NP groups; # NP SHR or P SHR versus NP Wistar rats.

Compared to endothelial cells of the aorta from NP Wistar rats, endothelial cells of the aorta from NP SHR showed reduced number of caveolae (Figures 3A,B). The aorta from P Wistar rats presented a smaller amount of endothelial caveolae than the aorta from NP Wistar rats. However, in SHR, pregnancy did not alter the number of caveolae in the aorta.

Treatment of the aorta with dextrin reduced the number of caveolae in NP Wistar rats, but it did not reduce the number of caveolae in the aorta from P Wistar rats (Figure 3C). Similarly, dextrin reduced the number of aortic endothelial caveolae in the aorta from NP SHR but not P SHR.

### Hypertension and Dextrin, but Not Pregnancy, Reduced the Number of Caveolae in the Resistance Mesenteric Artery From Rats

**Figure 4:**
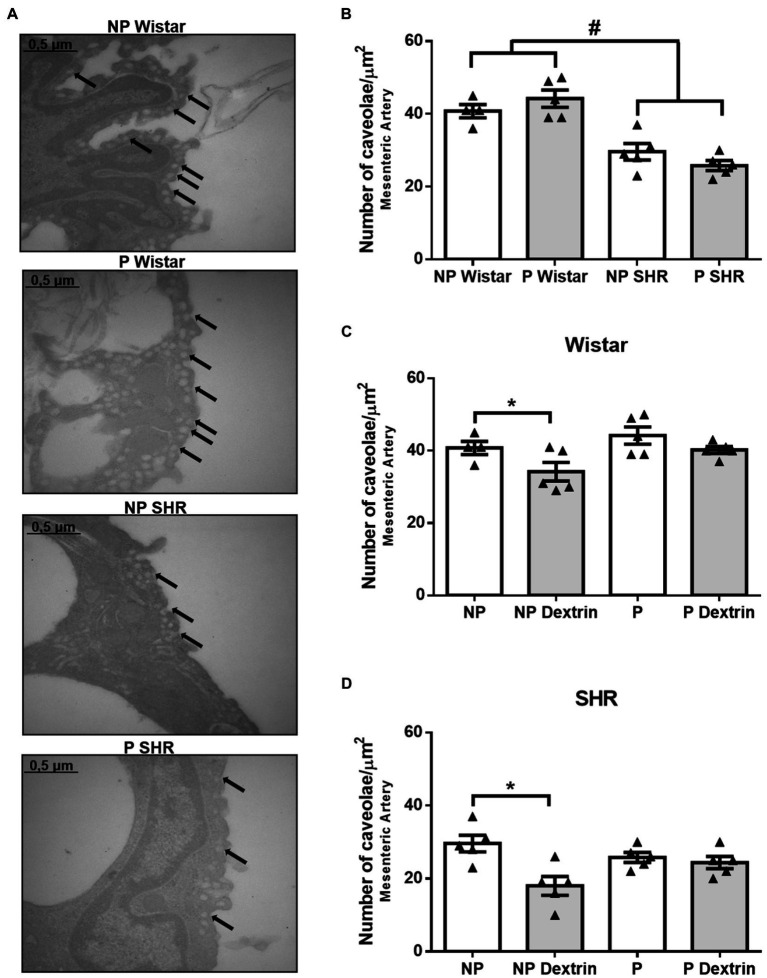
Representative photomicrographs (50,000× magnification, in **(A)** and quantification of the number of endothelial caveolae/μm^2^ in resistance mesenteric arteries of non-pregnant (NP) and pregnant (P) Wistar and spontaneously hypertensive (SHR) rats **(B)** in the presence of vehicle or dextrin (10mmol/L, **C,D**). The bars represent the mean±SEM of the results obtained in the resistance mesenteric artery (*n* =5) from the different groups. ^#^*p* <0.05 NP SHR and P SHR versus NP Wistar rats and P Wistar rats; ^*^ <0.05 NP dextrin versus NP.

Compared to the resistance mesenteric artery from NP Wistar rats and P Wistar rats, the resistance mesenteric artery from NP SHR and P SHR presented a reduced number of caveolae (Figures 4A,B). The number of endothelial caveolae in the resistance mesenteric artery from P Wistar rats was not statistically different from the number of endothelial caveolae in the resistance mesenteric artery from NP Wistar rats. In addition, there were no differences between the number of endothelial caveolae in the resistance mesenteric artery from NP SHR and P SHR (Figures 4A,B).

Dextrin reduced the number of caveolae in the resistance mesenteric artery from NP Wistar rats and NP SHR. However, dextrin did not alter the number of caveolae in the mesenteric artery from P Wistar rats or P SHR.

### Hypertension, but Not Pregnancy, Reduced Cav-1 and Phosphorylated Cav-1^Tyr14^ Expression in the Aorta From Rats

**Figure 5:**
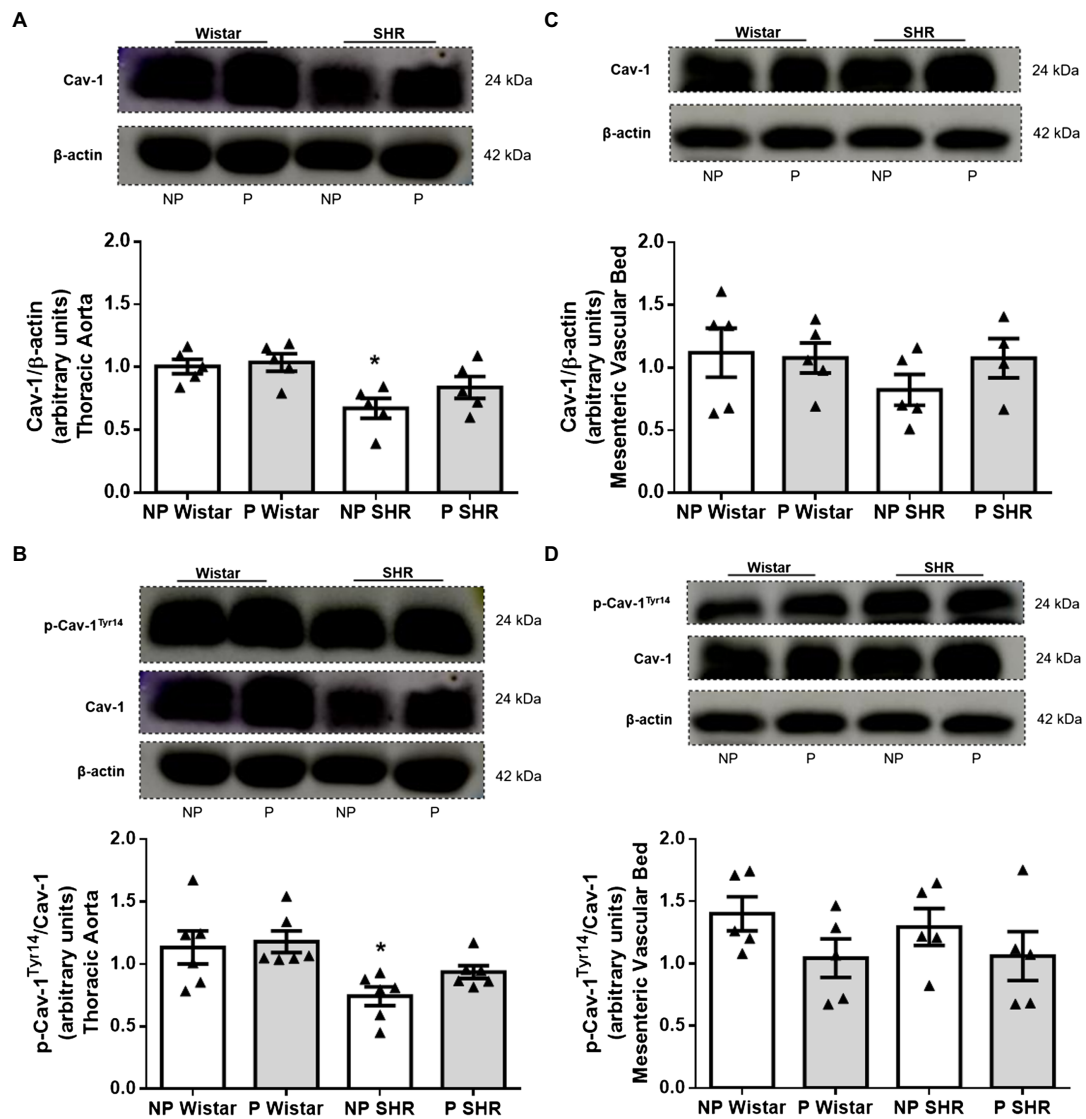
Representative images and quantification of the caveolin-1 (Cav-1)/β-actin **(A,C)** and p-Cav-1^Tyr14^/Cav-1 **(B,D)** expression in the aorta and resistance mesenteric artery homogenates of non-pregnant (NP) and pregnant (P) Wistar and spontaneously hypertensive (SHR) rats. The bars represent the mean±SEM of the results obtained in the aorta (*n*=5) from the different groups. ^*^p<0.05 NP SHR versus NP Wistar rats and P Wistar rats.

Compared to NP Wistar rats, Cav-1 expression was lower in aortic homogenates from NP SHR (Figure 5A). Still compared to NP Wistar rats, Cav-1 expression did not change in aortic homogenates from P Wistar rats (Figure 5A). Aortic homogenates from P SHR and NP SHR did not have statistically different Cav-1 expression (Figure 5A).

Compared to NP Wistar rats, the phosphorylated Cav-1^Tyr14^/total Cav-1 ratio was reduced in aortic homogenates from NP SHR. Pregnancy did not modify p-Cav-1^Tyr14^ expression in aortic homogenates from Wistar rats or SHR (Figure 5B). The mesenteric bed homogenates from the groups did not differ in terms of Cav-1 expression (Figure 5C) or p-Cav-1^Tyr14^ expression (Figure 5D).

### Reduced CaM Expression Was Observed in the Aorta, but Not in the Mesenteric Bed From SHR and Pregnancy Did Not Alter CaM Expression

**Figure 6:**
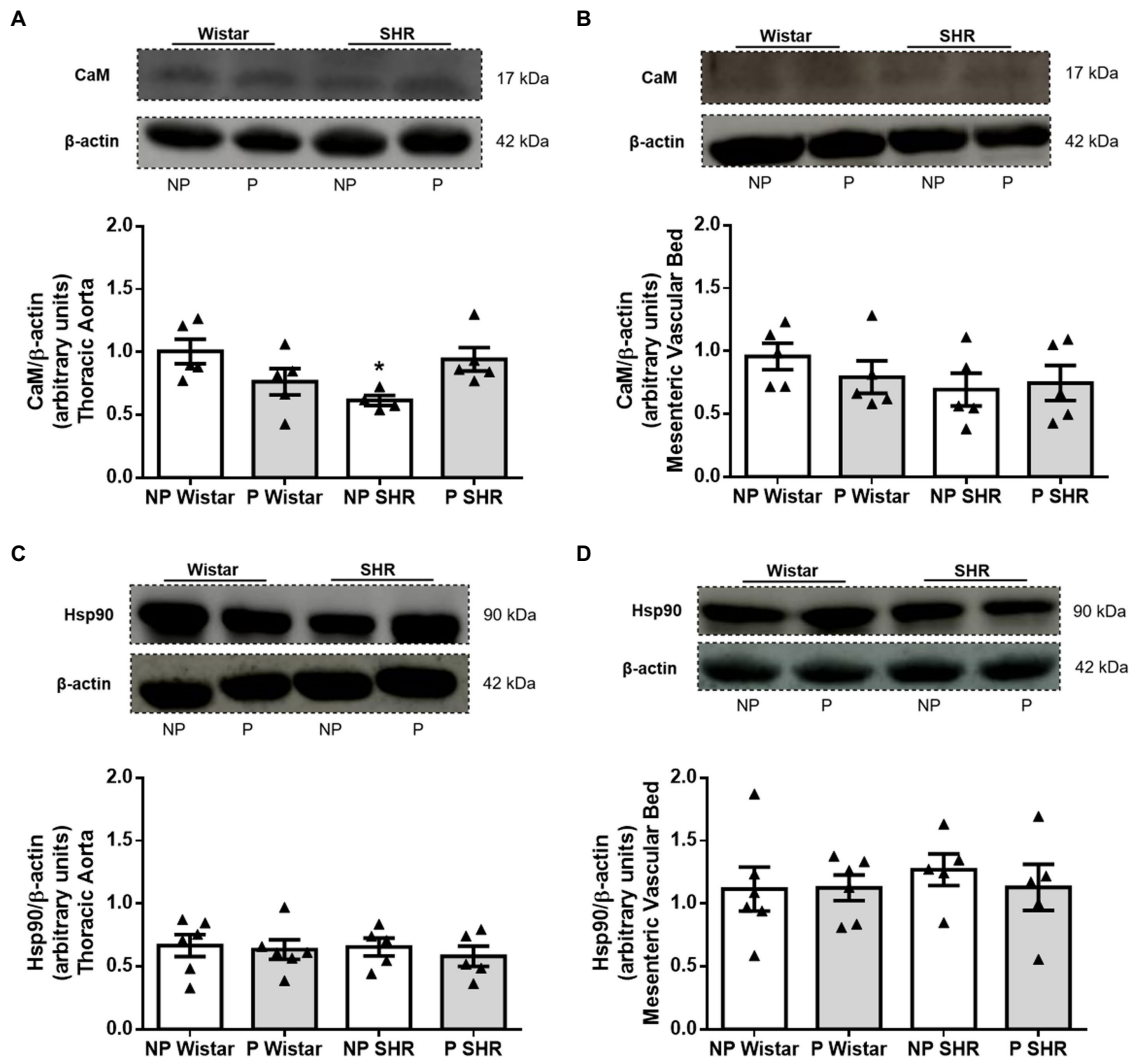
Representative images and quantification of calmodulin (CaM)/β-actin and heat shock protein 90 (Hsp90)/β-actin expression in the aorta (in **A,C**) and resistance mesenteric bed (in **B,D**) homogenates from non-pregnant (NP) and pregnant (P) Wistar and spontaneously hypertensive (SHR) rats. The bars represent the mean±SEM of the results obtained in the aorta and resistance mesenteric bed (*n*=5–6) from the different groups. ^*^p<0.05 NP SHR versus NP Wistar rats.

Compared to NP Wistar rats, CaM expression was decreased in aortic homogenates from NP SHR (Figure 6A). Pregnancy did not change CaM expression in aortic homogenates from Wistar rats or SHR (Figure 6A). CaM expression in mesenteric bed homogenates was not different in Wistar rats and SHR, and pregnancy did not change expression of this target protein (Figure 6B).

### Neither Hypertension nor Pregnancy Altered Hsp90 Expression in the Aorta or Mesenteric Bed From Wistar Rats or SHR

Compared to NP Wistar rats, Hsp90 protein expression was not altered in blood vessels from NP SHR (Figures 6C,D). Hsp90 expression was similar in aortic homogenates from NP Wistar rats and P Wistar rats (Figure 6C). Pregnancy did not modify Hsp90 expression in mesenteric bed homogenates (Figure 6D) from Wistar rats and SHR.

### Cav-1/eNOS Interaction Is Reduced by Hypertension and Pregnancy in Female Rat Blood Vessels

**Figure 7:**
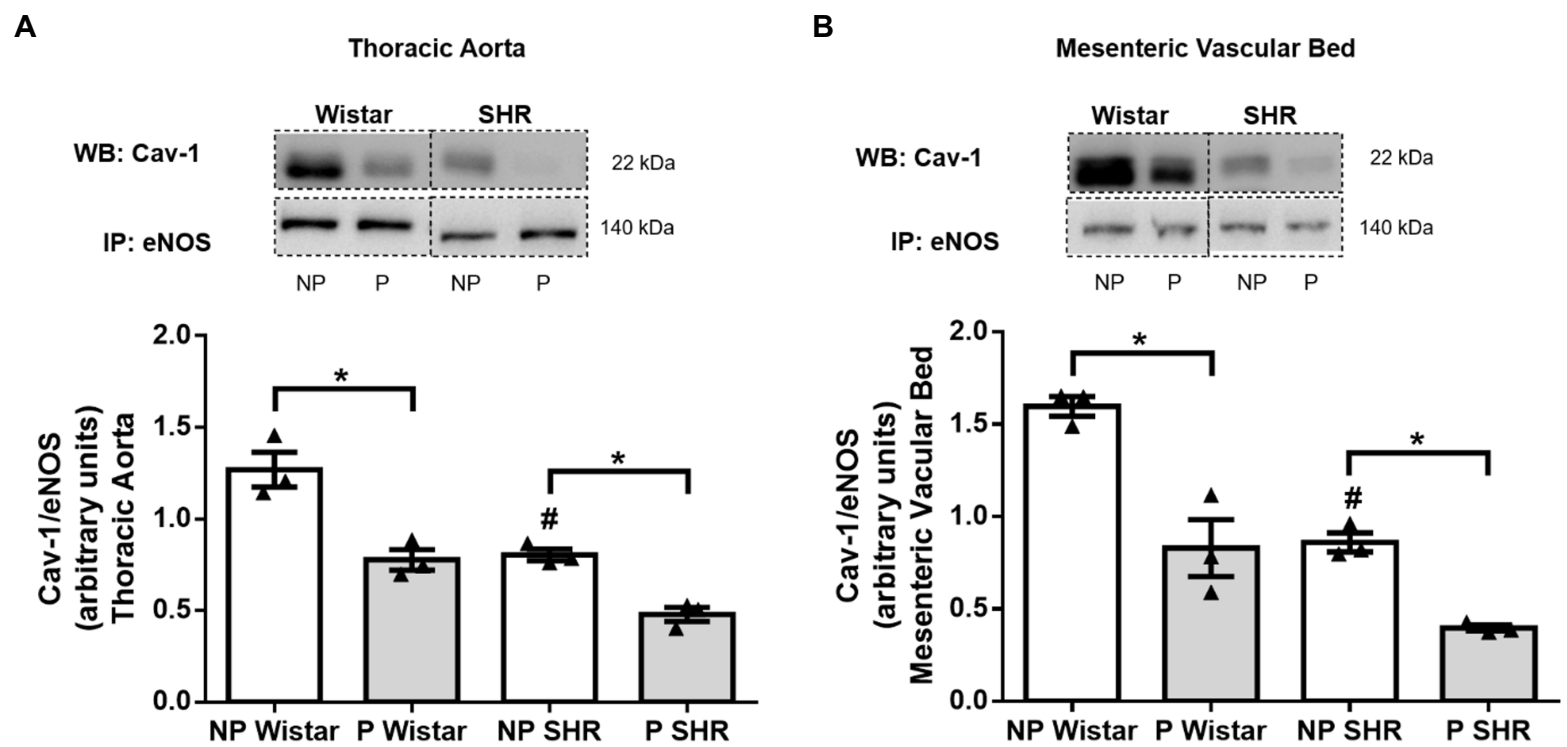
Analysis of Cav-1/eNOS interaction by co-immunoprecipitation (Co-IP) of endothelial nitric oxidase synthase (eNOS) associated with caveolin-1 (Cav-1) immunoblot (WB) in the aorta **(A)** and resistance mesenteric bed **(B)** of non-pregnant (NP) and pregnant (P) Wistar and spontaneously hypertensive (SHR) rats. The bars represent the mean±SEM of the results obtained in pools of the aorta and mesenteric bed (*n*=3) from the different groups. ^*^p<0.05 P versus NP groups; ^#^*p*<0.05 NP SHR versus NP Wistar rats.

Compared to NP Wistar rats, Cav-1/eNOS interaction in aortic and mesenteric vascular bed homogenates decreased in NP SHR (Figures 7A,B). Pregnancy reduced Cav-1/eNOS interaction in the aorta (Figure 7A) and mesenteric vascular bed (Figure 7B) from Wistar rats and SHR.

## Discussion

Caveolae are an important structure in vascular cells AND most eNOS is in these invaginations of the plasma membrane. Within the plasma membrane, eNOS is highly expressed in caveolae, and its activity is seven times greater in the plasma membrane than in the cytosol. eNOS activity is not detected in membrane fractions without caveolae. Furthermore, in resting conditions, most functional eNOS is in the caveolae, where between 57 and 100% eNOS activity is detected (Shaul et al., 1996). Accordingly, caveolae contain specific proteins that participate in eNOS signaling (Li et al., 1995; Chini and Parenti, 2004; Wang et al., 2005; Bernatchez et al., 2011).

We observed greater amount of NO in the aorta and mesenteric bed at the end of pregnancy, as indirectly measured by nitrite levels (Figures 1A,B). This increase in NO may be associated with increased total eNOS and p-eNOS^Ser1177^ expression in the aorta and higher NO concentration in aortic endothelial cells from P Wistar rats and P SHR, as already observed by our research team (Zancheta et al., 2015; Troiano et al., 2016). Moreover, in the aorta, where NO is the main regulatory factor of vascular homeostasis, variation of NO levels is greater in NP SHR and P SHR than in NP Wistar rats and P Wistar rats, emphasizing the importance of this molecule for regulation of vascular mechanisms during pregnancy of hypertensive rats.

To evaluate the role caveolae play in PE-induced contraction and ACh-induced relaxation, we performed functional reactivity studies on the aorta from NP Wistar rats, P Wistar rats, NP SHR, and P SHR in the presence of dextrin, a drug that disrupts the caveolae structure (Linder et al., 2005). As expected, dextrin disrupts the caveolae structure, compromising the eNOS function and favoring contractile effects, thereby increasing PE reactivity in the aorta from NP Wistar rats and NP SHR (Figures 2A,B). Dextrin increases PE-stimulated vascular contraction by a mechanism associated with lower eNOS-derived NO production in aortic rings (Potje et al., 2019) and femoral arteries from Wistar rats (Al-Brakati et al., 2015). In our study, dextrin shifted the concentration-responses curves to ACh to the right, and a lower magnitude of relaxation is observed only in vessels from NP Wistar rats and NP SHR (Figures 2C,D). These data reinforce the importance of stable caveolae for eNOS localization and activity in blood vessels from normotensive and hypertensive rats. Corroborating our data, dextrin impairs ACh-induced vasodilation in male normotensive rats, such as Sprague–Dawley (Linder et al., 2005) and Wistar (Rodrigues et al., 2009; Potje et al., 2019), and also in male hypertensive rats models, such as SHR (Potje et al., 2019) and two kidneys, one clip (2K-1C) (Rodrigues et al., 2009).

Interestingly, dextrin has no effects on PE contraction or ACh relaxation in the aorta from P Wistar rats or P SHR, suggesting that eNOS activity is less dependent on caveolae stability during pregnancy. Rizzo and collaborators (Rizzo et al., 1998) showed that increased vascular flow and pressure generates hemodynamic forces *in situ* that activate caveolar eNOS, thus dissociating eNOS from caveolin and favoring its association with CaM at the luminal endothelial cell surface, but not in caveolae plasma membrane. Additionally, after prolonged agonist stimulation, eNOS undergoes depalmitoylation and translocates to specific interior cell compartments or the cytosol (Michel et al., 1997; Yeh et al., 1999). These studies demonstrated that active eNOS is not present in caveolae structure, reinforcing our hypothesis that eNOS function is less dependent on caveolae stability during pregnancy. Besides that, higher NO production observed in arteries from pregnant rats (Figure 1) confirms increased eNOS activity during pregnancy.

We further investigated whether pregnancy alters the number of caveolae present in the endothelium of the aorta and resistance mesenteric artery from Wistar rats and SHR. By using electron microscopy, we observed that, compared to P-Wistar rats, the number of endothelial caveolae in the aorta and mesenteric artery from P SHR is reduced (Figures 3B, 4B). These results corroborate previous findings by our group of reduced number of caveolae in the aorta and mesenteric artery of male SHR compared to matched arteries in male Wistar rats (Potje et al., 2019). Compared to normotensive rats, male hypertensive 2K-1C rats also exhibit reduced number of aortic endothelial and smooth cell caveolae (Rodrigues et al., 2007; Rodrigues et al., 2009). Together, these results show that hypertension is associated with reduced amount of caveolae in arteries, which contributes to vascular dysfunction.

In Wistar rats, pregnancy significantly reduces the number of aortic endothelial caveolae (Figure 3B), but this effect seems to depend on the arterial bed because we did not observe it in the mesenteric artery (Figure 4B). Pregnancy does not change the caveolae density in the aorta or resistance mesenteric artery of P SHR (Figures 3B, 4B) compared to NP SHR. As expected, dextrin reduces the number of caveolae only in arteries from NP Wistar rats. However, in arteries from P Wistar rats and P SHR, dextrin does not modify the number of caveolae (Figures 3C,D and 4C,D). These results reinforce the suggestion that endothelial caveolae in pregnant rat arteries are more resistant to the effects of dextrin. A higher dextrin concentration may be needed to produce the same effect observed in vessels from non-pregnant rats.

We evaluated possible changes in the expression of proteins that control eNOS activity in pregnant rat arteries. Compared to Wistar rats, Cav-1 expression is reduced in the aorta, but not in the mesenteric artery from SHR. This result reinforces data showing decreased Cav-1 expression in male SHR aorta (Piech et al., 2003; Sánchez et al., 2006; Vera et al., 2007; Cristofaro et al., 2012), which may account for reduction in the number of caveolae. Pregnancy does not alter Cav-1 expression in vessels from Wistar rats or SHR (Figures 5A,C), but reduced the caveolae density only in the aorta from Wistar rats. Therefore, changes in caveolae density are not essentially followed by alterations in Cav-1 expression as reported by our group (Potje et al., 2019). Phosphorylated Cav-1^Tyr14^ binds to eNOS and consequently inactivates the enzyme. However, this mechanism compensates for the increase in eNOS^Ser1177^ phosphorylation *via* NO/Src, that is, increased NO due to increased eNOS^Ser1177^ phosphorylation would activate p-Src, causing Cav-1^Tyr14^ phosphorylation, which in turn increases eNOS binding/inhibition (Chen et al., 2012). Compared to NP Wistar rats and P Wistar rats, Cav-1^Tyr14^ phosphorylation is reduced in the aorta from NP SHR, which may suggest that lower Cav-1 activity stems from lower NO production (Figure 5B).

Compared to NP Wistar rats, aortic CaM expression is reduced in NP SHR, corroborating previous results showing reduced CaM expression in male SHR compared to normotensive rats (Piech et al., 2003; Sánchez et al., 2006; Vera et al., 2007; Cristofaro et al., 2012). Pregnancy does not alter CaM expression in arteries from Wistar rats or SHR (Figures 5A,C). Different studies have shown that ACh-induced relaxation is not altered at the end of pregnancy in Wistar rats (Ballejo et al., 2002; Zancheta et al., 2015). These data suggest that pregnancy does not play a role in direct activation of the Ca^2+^-Calmodulin complex or changes in CaM expression.

Hsp90 binding to eNOS stimulates eNOS activity, increasing the catalytic function of the enzyme and maintaining balance between eNOS-derived NO and superoxide anion (Brouet et al., 2001; Pritchard et al., 2001). Experiments on human umbilical vein endothelial cells and isolated rat aorta have shown that eNOS activity increases when a complex between eNOS and Hsp90 is formed, increasing NO production (Wang et al., 2009). The link between Hsp90 expression and phosphorylated eNOS in male SHR aortic tissues is lower compared to normotensive rats (WKY), and this relationship increases when SHR are treated with sodium nitrite (Ling et al., 2016). To date, our results have shown that total Hsp90 expression remains unchanged in arteries from NP SHR arteries compared to arteries from NP Wistar rats (Figures 5B,D), suggesting that Hsp90 appears not to be involved in eNOS activation in arteries from pregnant rats. However, it has been demonstrated that Hsp90 subunits play a specific role in eNOS activity, with Hsp90α causing Akt and eNOS^Ser1177^ phosphorylation, increasing NO production; and with Hsp90β leading to eNOS^Thr495^ phosphorylation, producing superoxide anion (Tanaka et al., 2015). Further studies should be carried out to evaluate possible changes in Hsp90α expression and activity in arteries from pregnant rats.

We also analyzed Cav-1/eNOS interaction. As reported throughout this manuscript, decreased eNOS/Cav-1 interaction increases eNOS activity and NO production. eNOS/Cav-1 interaction is reduced in arteries from SHR compared to Wistar rats, which is followed by smaller amounts of NO in hypertensive vessels. Impaired NO production and signaling in hypertension has been attributed to eNOS uncoupling, which is associated with tetrahydrobiopterin (BH4) oxidation, L-arginine deficiency, eNOS S-glutathionylation, eNOS-dependent superoxide production, or increased NOX activity (Li et al., 2015). Therefore, less interaction in SHR is not associated with higher NO levels because eNOS may be uncoupled during hypertension. In addition, pregnancy further decreases eNOS/Cav-1 interaction in arteries from Wistar rats and SHR (Figures 7A,B), which may contribute to higher NO levels (Figure 1) and greater eNOS activity, as already shown (Zancheta et al., 2015). eNOS may translocates to other cell compartments, with its specific localization in caveolae not being mandatory at the end of pregnancy.

Taken together, our data demonstrated that eNOS function is less dependent on caveolae stability during pregnancy and that pregnancy does not alter the main eNOS regulatory proteins expression, but it decreases Cav-1/eNOS interaction. In conclusion, reduced Cav-1/eNOS interaction in the aorta and mesenteric vascular bed seems to be an important mechanism to increase eNOS activity and nitric oxide production in blood vessel from pregnant normotensive and hypertensive rats.

## Data Availability Statement

The original contributions presented in the study are included in the article/Supplementary Material, further inquiries can be directed to the corresponding author.

## Ethics Statement

The animal study was reviewed and approved by Animal Use Ethics Committee of the School of Dentistry, Sao Paulo State University (UNESP), Aracatuba, Sao Paulo, Brazil.

## Author Contributions

JT, SP, and CA conceived and designed the experiments. JT, SP, MG, and EG performed the experiments. JT, SP, RT, and CA wrote the manuscript. All authors contributed to the article and approved the submitted version.

## Funding

This work was supported by Fundação de Amparo à Pesquisa do Estado de São Paulo – FAPESP (grant numbers 2015/09373–0, 2016/22180–9, and 2018/10635–7) and Coordenação de Aperfeiçoamento de Pessoal de Nível Superior – CAPES (code 001).

## Conflict of Interest

The authors declare that the research was conducted in the absence of any commercial or financial relationships that could be construed as a potential conflict of interest.

The reviewer [SC] declared a past co-authorship with one of the authors [RT] to the handling editor.

## Publisher’s Note

All claims expressed in this article are solely those of the authors and do not necessarily represent those of their affiliated organizations, or those of the publisher, the editors and the reviewers. Any product that may be evaluated in this article, or claim that may be made by its manufacturer, is not guaranteed or endorsed by the publisher.
