# Supplementary figures and images for: Caveolin-1/Endothelial Nitric Oxide Synthase Interaction Is Reduced in Arteries From Pregnant Spontaneously Hypertensive Rats
Source: Front Physiol. 2021 Nov 9;12:760237. doi: 10.3389/fphys.2021.760237 (PMC8631196; doi:10.3389/fphys.2021.760237)

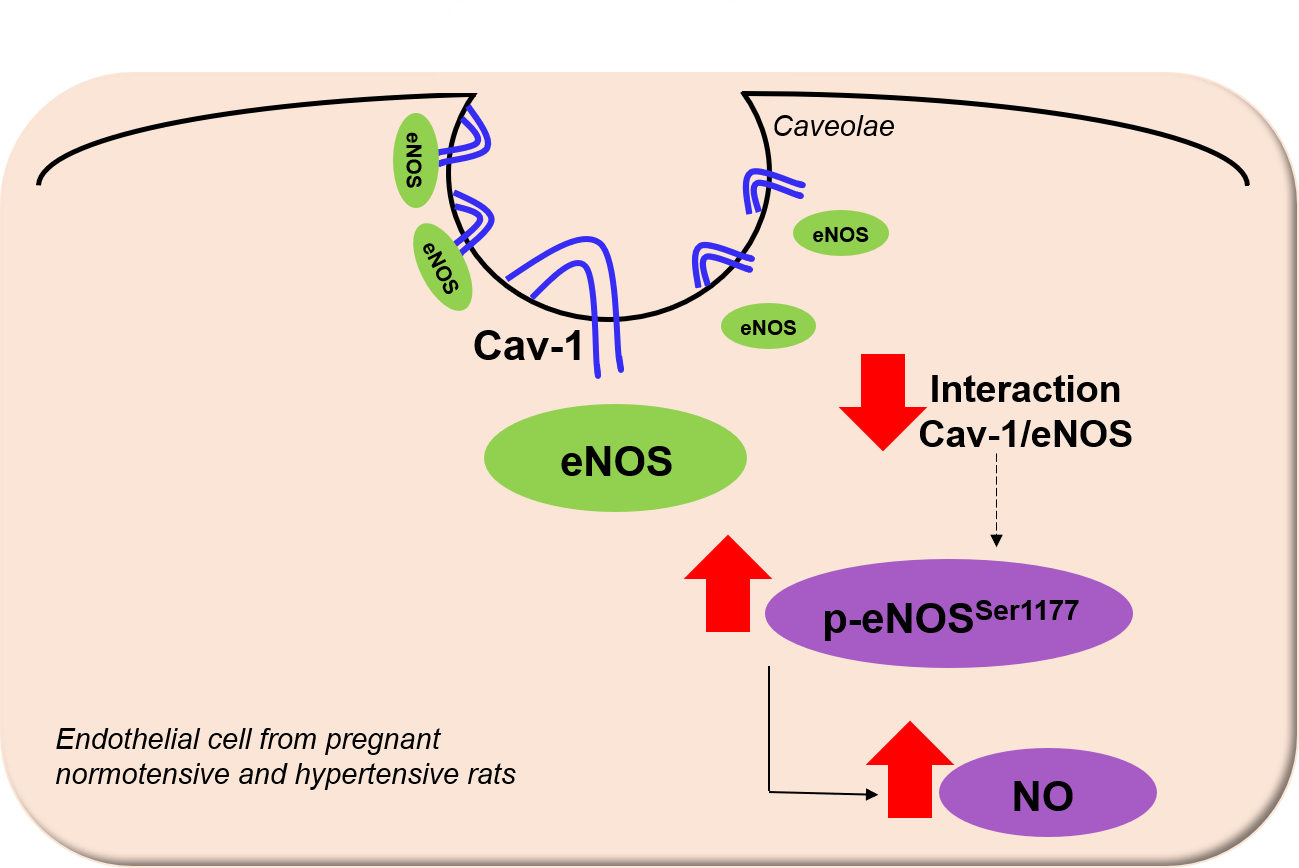

Supplement: Supplementary file 1 [file Image_1.TIF]
